# Influence of ferrule preparation with or without glass fiber post on fracture resistance of endodontically treated teeth

**DOI:** 10.1590/S1678-77572010000400007

**Published:** 2010

**Authors:** Alexandra Furtado de LIMA, Aloísio Oro SPAZZIN, Daniel GALAFASSI, Lourenço CORRER-SOBRINHO, Bruno CARLINI-JÚNIOR

**Affiliations:** 1 DDS, Graduate student, Department of Restorative Dentistry, Dental School, University of Passo Fundo, Passo Fundo, RS, Brazil.; 2 DDS, MSc, Graduate student, Department of Restorative Dentistry, Dental Materials Division, Piracicaba Dental School, State University of Campinas, Piracicaba, SP, Brazil.; 3 DDS, MSc, Graduate student, Department of Restorative Dentistry, Ribeirão Preto Dental School, University of São Paulo, Ribeirão Preto, SP, Brazil.; 4 DDS, MSc, PhD, Professor, Department of Restorative Dentistry, Dental Materials Division, Piracicaba Dental School, State University of Campinas, Piracicaba, SP, Brazil.; 5 DDS, MSc, PhD, Professor Department of Restorative Dentistry School of Dentistry, University of Passo Fundo, RS, Passo Fundo, RS, Brazil.

**Keywords:** Ferrule preparation, Glass fiber post, Fracture resistance

## Abstract

**Objective:**

This study evaluated the effect of ferrule preparation (Fp) on the fracture resistance of endodontically treated teeth, restored with composite resin cores with or without glass fiber posts.

**Material and Methods:**

Forty-four bovine teeth were sectioned 19 or 17 mm (2 mm ferrule) from the apex, endodontically treated and assigned to four groups (n = 11): Group 1: Fp and post; Group 2: Fp and without post; Group 3: without Fp and with post; Group 4: without Fp and without post. All specimens were restored with composite resin core and metal crown. Specimens were subjected to fracture resistance testing in a universal testing machine at a crosshead speed of 0.5 mm/min. The data were analyzed by two-way ANOVA and Tukey’s tests (α=0.05).

**Results:**

The mean fracture resistance values were as follows: Group 1: 573.3 N; Group 2: 552.5 N; Group 3: 275.3 N; Group 4: 258.6 N. Significantly higher fracture resistance was found for the groups with Fp (p<0.001).

**Conclusion:**

There was no statistically significant interaction between the "Fp" and "post" factors (p = 0.954). The ferrule preparation increased the fracture resistance of endodontically treated teeth. However, the use of glass fiber post showed no significant influence on the fracture resistance.

## INTRODUCTION

An endodontically treated tooth often has limited remaining tooth structure to provide retention for a definitive restoration, and the loss of root and coronal dentin increases its susceptibility to fracture^4^. Sound coronal dentin should be conserved during restorative procedures to extend the crown margin below the junction of the core and the remaining tooth structure^2^. However, the clinician often finds that horizontal loss of the clinical crown has occurred and that little ferrule can be created on the remaining tooth structure^10^. In these situations, there are few alternatives for restoration with a post-and-core buildup. The choice of an appropriate restoration for endodontically treated teeth is guided by strength and esthetics^8,23^.

The cast gold post and core has been regarded as the "gold standard" in post-and-core restorations due to its high success rate^3^. Alternatives to cast posts and cores have been developed. The use of prefabricated posts and custom-made buildups with composite has simplified the restorative procedure because all steps can be completed at the chairside, and fair clinical success can be expected^15^. The restoration of endodontically treated teeth using metal-free materials with physical properties similar to those of dentin has been suggested as an objective in restorative dentistry^20,22^. Manocci et al.^11^ (1999) investigated the intermittent loading response of teeth restored with quartz-fiber, carbon-quartz fiber, and zirconium posts and concluded that the fiber posts were able to reduce the risk of root fractures.

Posts have been thought to strengthen the remaining tooth structure, but this opinion has changed, and the belief that a tooth is strengthened by the incorporation of a post has been rejected^19^. The influence of the ferrule preparation^1,2,9,12,13^ and different post systems^11^ has been evaluated, but there is limited information about the effects of the ferrule preparation and composite resin core with or without use of glass fiber post. Thus, the aim of this study was to evaluate the influence of a 2-mm ferrule preparation and use of glass fiber post on the fracture resistance of endodontically treated teeth restored with composite cores and crowns. The null hypothesis was that the variables investigated had no influence on the fracture resistance.

## MATERIAL AND METHODS

A total of 44 teeth, selected from a initial sample of 420 bovine incisors, were used in the study. They were stored in a solution of water and 0.1% thymol at room temperature. A digital caliper (Mitutoyo, Suzano, SP, Brazil) was used to measure the mesiodistal and buccolingual dimensions of each tooth at the cementoenamel junction to ensure the distribution of teeth of similar size included in the study groups. The teeth were scaled to remove organic debris and stored in physiologic solution at 4°C.

**Figure 1:**
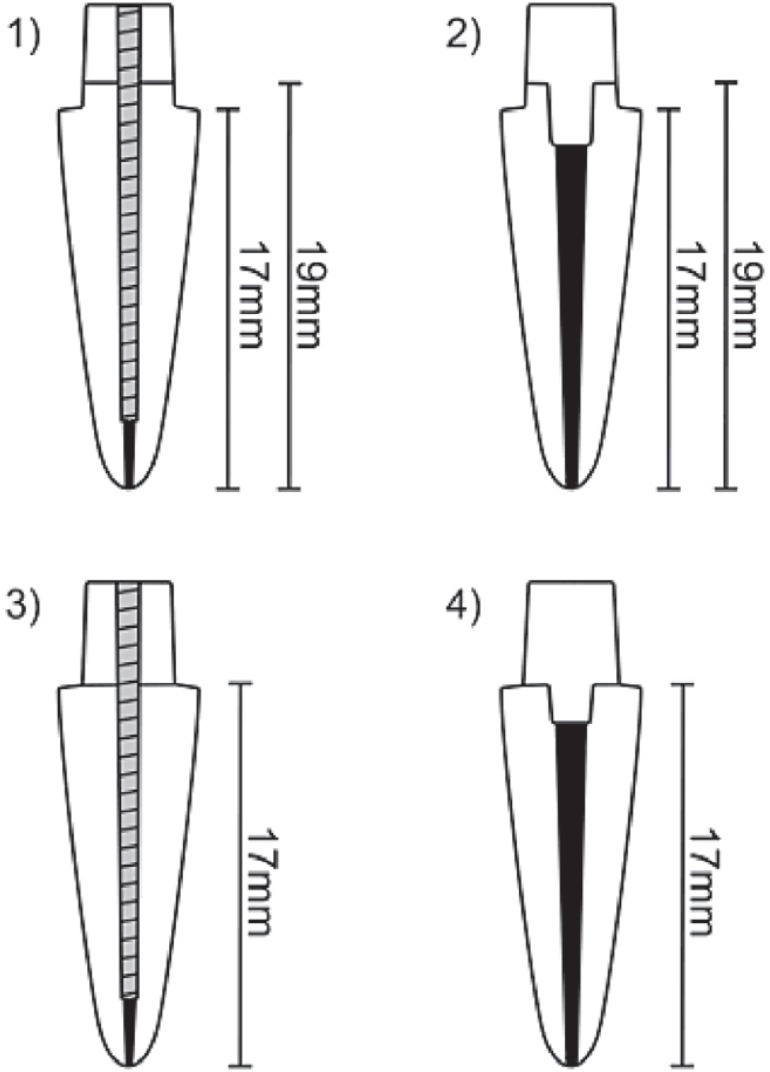
Specimen designs in the different groups: (1) with Fp and with post; (2) with Fp and without post; (3) without Fp and with post; and (4) without Fp and with post

Crowns were removed below the cementoenamel junction to obtain a root length of 19 and 17 mm (2 mm was used as ferrule preparation). The roots were endodontically treated. After endodontic instrumentation, all canals were filled by lateral condensation of gutta-percha (Dentsply-Maillefer, Petrópolis, RJ, Brazil) and root canal sealer (Endofill; Dentsply-Maillefer). The prepared roots were randomly divided into four groups (n = 11), according to the canal and core restoration. Schematic representations of the groups are shown in Figure 1.

In Groups 1 and 3, the root canal preparations were made with a #2 reamer (Largo; Dentsply-Maillefer) to remove 13 and 11 mm of gutta-percha, respectively, leaving approximately 5 mm of canal filling at the apex. The posts (#2 Reforpost; Angelus, Londrina, PR, Brazil) were cemented with resin cement (RelyX ARC; 3M eSPe, St. Paul, MN, USA), using an etch-and-rinse adhesive system (Scotchbond Multi-Purpose; 3M eSPe) in accordance with the manufacturer’s directions. For core fabrication, the dentin was etched with 37% phosphoric acid (Dentsply), and a one-bottle adhesive system (Primer Bond 2.1; Dentsply) was applied to the dentin as recommended by the manufacturer. The composite resin cores were standardized using a core-forming matrix (TDV Dental Ltd, Pomerode, SC, Brazil) and a composite resin (Z250; 3M ESPE). The composite resin was placed using an incremental technique, each increment requiring 40 s of polymerization (Optilux VCL401; Demetron-Kerr, Danbury, Conn, USA) to complete the coronal core. The tip of the light-curing unit was positioned 1 cm from the specimens at the top of the core.

In Groups 2 and 4, the gutta-percha was removed (4 and 2 mm, respectively) and root canal entrances were enlarged with a tapered diamond bur (#4138, KG Sorensen, Barueri, SP, Brazil). The prepared root canals were filled with composite resin only and the core made in the same way as for Groups 1 and 3.

All specimens were prepared with a diamond bur (#3216; KG Sorensen) in a high-speed handpiece with water spray cooling. Specimens were prepared to receive complete crowns (1.5 mm facial reduction with a chamfer finish line and 0.5 mm chamfered lingual reduction). The chamfer finish line was located 17 mm from the root apex. The full crowns were fabricated of acrylic resin (Duralay; Reliance Dental Mfg. Co., Chicago, USA) to serve as a pattern for casting with a Ni-Cr metal alloy. The crowns shapes were standardized with prefabricated acrylic resin crowns. All metal crowns were cemented with zinc phosphate cement (SS White, Rio de Janeiro, RJ, Brazil).

**Figure 2:**
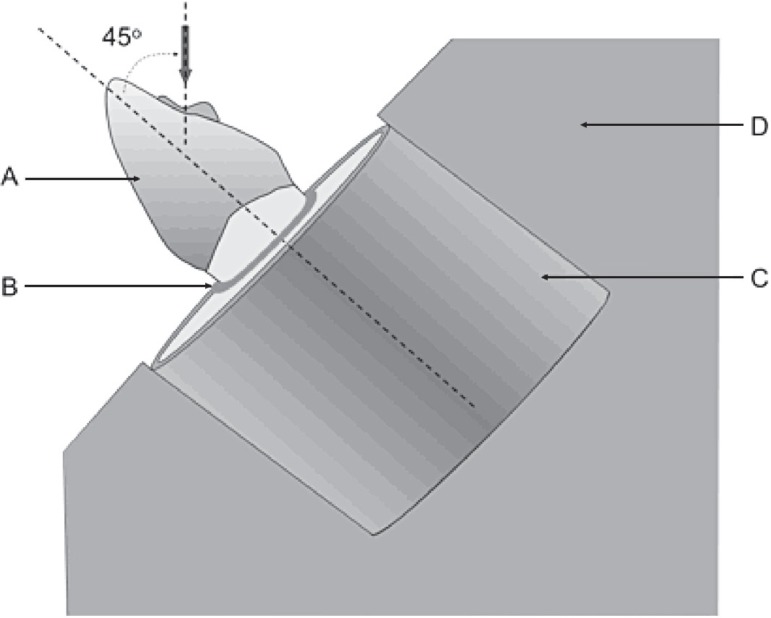
Schematic drawing of the specimen mounted in an acrylic resin block; (A) metal crown; (B) silicon-simulated periodontal ligament; (C) resin block; (D) metal device. Arrow indicates 45° angle of load applied to prepared notch on the palatal surface

The root surface of each tooth was coated with a layer (approximately 60 µm) of elastomeric impression material (Aquasil; Dentsply-DeTrey, Konstanz, Germany) to simulate a periodontal ligament^12,18^. Specimens were submitted to the fracture resistance testing using a universal testing machine (eMIC DL-2000, eMIC, São José dos Pinhais, PR, Brazil). A compressive load was applied on the lingual surface (2 mm below the incisal edge) at a 45-degree angle and crosshead speed of 0.5 mm/min until a fracture occurred (Figure 2)^21,23^. The load was applied with a 2-mm-diameter ball-shaped metallic advice. Fracture resistance values (N) and fracture patterns were recorded in N. Two-way ANOVA was used to compare the mean loads for each group. The dependent variable was the load required to fracture the specimens. A significant ANOVA result was followed by the Tukey’s test (α=0.05).

## RESULTS

The fracture resistance results of all groups are reported in Table 1. The two-way ANOVA followed by the Tukey’s test showed that the factor "post" did not significantly interfere with fracture resistance (*p* = 0.633). Groups 1 and 3 did not present significantly higher fracture resistance than Groups 2 and 4. On the other hand, the factor "ferrule preparation" was significant (*p* ≤ 0.001), since its presence in Groups 1 and 2 resulted in significantly higher fracture resistance values than those in Groups 3 and 4 (p < 0.001). There was no significant interaction between "post" and "ferrule preparation" (p = 0.954). The placement of a post showed no influence, irrespective of the presence of ferrule preparation. The fracture pattern was similar for all groups. The failures occurred in the level of the crown-dentin interface or cervical third of the root. All failures were considered restorable with association or not of periodontal surgery.

**Tabela 1:** Means and standard deviations of fracture resistance (N) for the four groups

**Group**	**n**	Fracture Resistance *
		
1	11	573.9 (153.9) a
2	11	552.5 (126.9) a
3	11	275.3 (62.4) b
4	11	258.6 (109.8) b

* Means followed by different lowercase letters (column) are statistically different, Tukey's test (p<0.05).

## DISCUSSION

Based on the results of the study, the null hypothesis that variation of the parameters would not influence fracture resistance was partially rejected, since the ferrule preparation improved the fracture resistance, regardless of whether the composite resin core was associated with or without a glass fiber post. Previous studies have confirmed that crowned and endodontically treated teeth are subject to stress in the cervical region, and that a cervical ferrule preparation creates a positive effect on reducing stress concentration at the core-dentin junction^14^. The positive effect of a ferrule preparation on resistance to fracture has been extensively demonstrated^1,2,9,12^. Conversely, Pereira, et al.^13^ (2005) reported that the ferrule preparation decrease the fracture resistance. However, in that study, the teeth were subjected to the fracture resistance test without use of crowns and thus the effect of the ferrule preparation might have been masked. A study using finite element analysis could elucidate this doubt.

In addition, the ferrule preparation helps to maintain the integrity of the cement seal of the crown. Libman and Nicholls^10^ (1995) evaluated the *in vitro* effects of ferrules on the integrity of the cement seal of cast crowns and reported improved resistance to fatigue failure of the cement seal of a crown, when the crown margin extended at least 1.5 mm apical to the margin of the core. Moreover, when the ferrule is absent or extremely small, occlusal loads may cause the post to flex with eventual micromovement of the core, and the cement seal at the margin of the crown may fracture in a short time with resultant leakage and caries^7^.

When a glass fiber post was used, the fracture resistance mean was found to be a little higher, although the increase in fracture resistance in these groups was not found to be statistically significant when compared to the groups in which the glass fiber post was not used. The findings of the present study is in agreement with those of some studies using posterior teeth^6,17^. Salameh, et al.^17^ (2006) compared the fracture resistance of mandibular molars restored with resin composites by the direct technique, with or without fiber posts. They concluded that the resistance is mainly affected by the number of residual walls, and the presence or absence of glass fiber post did not affect the fracture resistance. Another study evaluating the fracture resistance of endodontically treated premolars also found that posts do not improve the fracture resistance^6^. In addition, Sahafi, et al.^16^ (2005) concluded that the use of a post did not increase the resistance to cyclic loading when compared to the use of a resin composite core alone.

A study using finite element analysis showed that the absence of a cervical ferrule was a determinant negative factor, giving rise to considerably higher stress levels. In addition, the authors related that if a ferrule was available, the choice of reconstruction material had no impact on the level of cervical stress. When a carbon fiber post was used without ferrule preparation, cervical stress levels were lower than they were when no post was used^14^. These data could explain the increase in fracture resistance of the teeth with a ferrule preparation. Nevertheless, post placement was not able to significantly increase the fracture resistance.

The mean fracture resistance values found for all groups were higher than the maximum bite force in the anterior teeth of men and women. Ferrario, et al.^5^ (2004) reported single-tooth bite forces in healthy young men and women ranging from 75 to 190 N in anterior teeth. The presence of a ferrule preparation was shown to enhance the fracture resistance of endodontically treated teeth with composite core and crown, irrespective of the use of a glass fiber post. Although the results of the present study did not show a significant improvement in fracture resistance with the use of a glass fiber post associated with a composite core, it would be precipitated to state that the posts are not necessary. Thus, further research is still necessary to investigate the longevity of these restorations, especially under clinical conditions.

## CONCLUSIONS

The following conclusions can be drawn from this *in vitro* study:

The presence of a ferrule preparation was shown to increase the fracture resistance of endodontically treated teeth with composite core and crown, irrespective of whether or not a glass fiber post was placed;

The use of a glass fiber post associated with a composite core showed no significant influence on the fracture resistance of endodontically treated teeth, irrespective of the presence of a ferrule preparation.
